# Leading determinants of incident dementia among individuals with and without the apolipoprotein E ε4 genotype: a retrospective cohort study

**DOI:** 10.1186/s12883-024-03557-8

**Published:** 2024-02-20

**Authors:** Siting Ye, Eddy Roccati, Wei Wang, Zhuoting Zhu, Katerina Kiburg, Yu Huang, Xueli Zhang, Xiayin Zhang, Jiahao Liu, Shulin Tang, Yijun Hu, Zongyuan Ge, Honghua Yu, Mingguang He, Xianwen Shang

**Affiliations:** 1 The Second Clinical College of Guangzhou University of Chinese Medicine, Guangzhou, 510405 China; 2 Department of Ultrasound, The Second Affiliated Hospital of Guangzhou University of Chinese Medicine, Guangzhou, 510120 China; 3 Guangdong Eye Institute, Department of Ophthalmology, Guangdong Provincial People’s Hospital (Guangdong Academy of Medical Sciences), Southern Medical University, Guangzhou, 510080 China; 4 Guangdong Cardiovascular Institute, Guangdong Provincial People’s Hospital, Guangdong Academy of Medical Sciences, Guangzhou, 510080 China; 5 Centre for Eye Research Australia, Melbourne, VIC 3002 Australia; 6 Department of Medicine (Royal Melbourne Hospital), University of Melbourne, Melbourne, VIC 3050 Australia; 7 Wicking Dementia Research and Education Centre, University of Tasmania, Hobart, TAS 7001 Australia; 8 State Key Laboratory of Ophthalmology, Zhongshan Ophthalmic Center, Sun Yat-Sen University, Guangzhou, 510060 China; 9 Monash e-Research Center, Faculty of Engineering, Airdoc Research, Nvidia AI Technology Research Center, Monash University, Melbourne, VIC 3800 Australia

**Keywords:** Dementia, Alzheimer disease, Apolipoproteins E, Life style, Multimorbidity risk score, Biomarker

## Abstract

**Background:**

Little is known regarding the leading risk factors for dementia/Alzheimer’s disease (AD) in individuals with and without APOE4. The identification of key risk factors for dementia/Alzheimer’s disease (AD) in individuals with and without the APOE4 gene is of significant importance in global health.

**Methods:**

Our analysis included 110,354 APOE4 carriers and 220,708 age- and sex-matched controls aged 40–73 years at baseline (between 2006–2010) from UK Biobank. Incident dementia was ascertained using hospital inpatient, or death records until January 2021. Individuals of non-European ancestry were excluded. Furthermore, individuals without medical record linkage were excluded from the analysis. Moderation analysis was tested for 134 individual factors.

**Results:**

During a median follow-up of 11.9 years, 4,764 cases of incident all-cause dementia and 2065 incident AD cases were documented. Hazard ratios (95% CIs) for all-cause dementia and AD associated with APOE4 were 2.70(2.55–2.85) and 3.72(3.40–4.07), respectively. In APOE4 carriers, the leading risk factors for all-cause dementia included low self-rated overall health, low household income, high multimorbidity risk score, long-term illness, high neutrophil percentage, and high nitrogen dioxide air pollution. In non-APOE4 carriers, the leading risk factors included high multimorbidity risk score, low overall self-rated health, low household income, long-term illness, high microalbumin in urine, high neutrophil count, and low greenspace percentage. Population attributable risk for these individual risk factors combined was 65.1%, and 85.8% in APOE4 and non-APOE4 carriers, respectively. For 20 risk factors including multimorbidity risk score, unhealthy lifestyle habits, and particulate matter air pollutants, their associations with incident dementia were stronger in non-APOE4 carriers. For only 2 risk factors (mother’s history of dementia, low C-reactive protein), their associations with incident all-cause dementia were stronger in APOE4 carriers.

**Conclusions:**

Our findings provide evidence for personalized preventative approaches to dementia/AD in APOE4 and non-APOE4 carriers. A mother’s history of dementia and low levels of C-reactive protein were more important risk factors of dementia in APOE4 carriers whereas leading risk factors including unhealthy lifestyle habits, multimorbidity risk score, inflammation and immune-related markers were more predictive of dementia in non-APOE4 carriers.

**Supplementary Information:**

The online version contains supplementary material available at 10.1186/s12883-024-03557-8.

## Introduction

Dementia was the fifth leading cause of death accounting for 2.4 million deaths globally in 2016 [1]. Age is the greatest risk factor for dementia with a prevalence of 5.0%, 13.1%, and 33.2%, respectively, among people aged 65–74, 75–84, and ≥ 85 years [2]. Given the increasing ageing population worldwide, the number of people with dementia is estimated to grow substantially in the future [3, 4]. As there are no effective ways to prevent or delay symptom progression yet, it is critical to identify important modifiable determinants for dementia [4–6].

Therapeutic options remain to target the symptoms of dementia/Alzheimer’s disease (AD), while no drugs have been approved for stopping the progression or even reversal of the disease [7]. The APOE gene has been convinced with high association with AD. The APOE gene has three common alleles: APOE2, APOE3, and APOE4. These alleles determine the structure of the apolipoprotein E protein, which plays a role in lipid metabolism and transportation in the body. Apolipoprotein E ε4 (APOE4) is the strongest single genetic risk factor for the development of AD. On the other hand, the APOE2 allele has been associated with a potentially protective effect against AD [8]. Research suggests that individuals with the APOE2 allele may have a reduced risk of developing the disease compared to those with the APOE3 or APOE4 alleles [9]. The APOE3 allele is the most common variant of the APOE gene. In the context of dementia, particularly AD, the APOE3 allele is considered the neutral or average risk allele. Unlike the APOE4 allele, which is a well-established genetic risk factor for AD, the APOE3 allele does not significantly increase or decrease the risk of developing dementia. It is estimated that APOE4 accounted for 53% (population attributable risk [PAR]) of AD overall with 70% among individuals aged 65–70 years [10]. Subsequently, APOE4 has been thought to be an emerging therapeutic target for AD [11]. Dementia is now recognized as a complex interplay between genetic and environmental factors [12]. Therefore, investigating the association between environmental factors, such as risk factors and lifestyle, with genotype has become a matter of utmost importance [13]. Testing gene-environment interactions in dementia provides the potential to explore more personalized preventative approaches for dementia among at-risk individuals [7]. Several cohort studies have shown that the association between higher physical activity and better sleep consolidation and lower risk of dementia was stronger among APOE4-carriers [14, 15]. Another cohort study has demonstrated that exposure to air pollution may have a greater potential impact on pathological brain aging in APOE4 carriers than in non-APOE4 carriers [16]. An interplay between APOE4 and plasma markers in AD has also been examined in a previous study [17]. However, it is unclear whether associations between a wide range of determinants and incident dementia differ between APOE4 and non-APOE4 carriers.

Although numerous risk factors have been linked to dementia [4, 18], little is known regarding the leading determinants in individuals with different genetic risks. It is important to test the interaction between individual determinants and APOE4 in the development of dementia/AD for designing personalized preventative strategies. Using the UK Biobank, we sought to examine associations between a wide range of risk factors and incident all-cause dementia/AD in individuals with and without APOE4, and whether APOE4 modified these associations.

## Methods

**Fig. 1:**
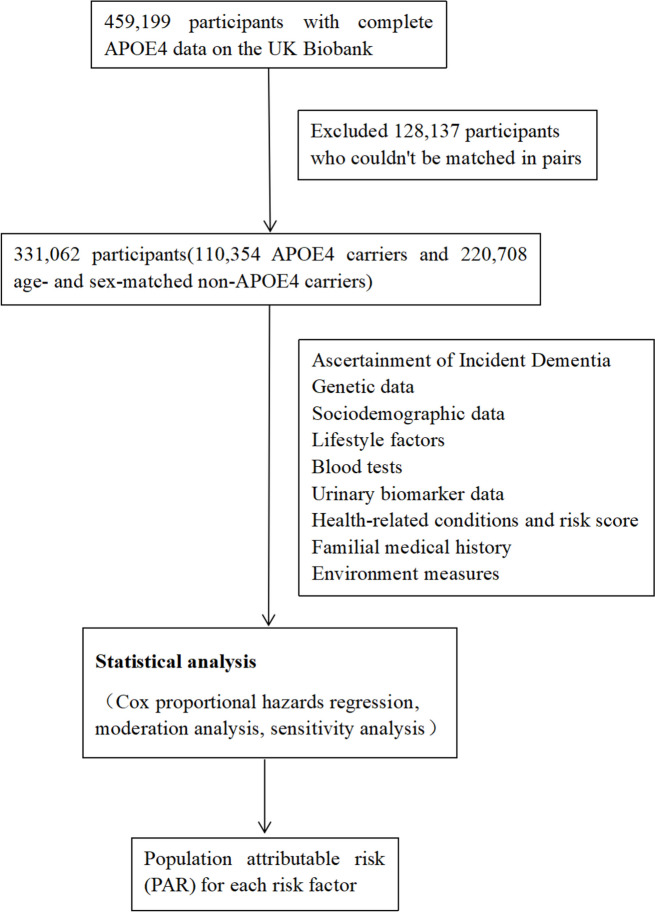
Workflow for this study

The workflow of this study has been presented in Fig. 1.

### Study population

This analysis was based on the UK Biobank, which is a population-based cohort of more than 500,000 participants aged 40–73 years old with baseline data collected between 2006–2010 [19]. We included all the participants with complete health records. We excluded individuals of non-European ancestry, those who couldn’t be linked to inpatient data, those with prevalent dementia, cognitive impairment, and those who developed dementia within the first year of follow-up. Among the initial 459,199 participants with complete APOE4 data, we further excluded 128,137 participants who couldn’t be matched in pairs. The final analysis included 331,062 participants (110,354 APOE4 carriers and 220,708 age- and sex-matched non-APOE4 carriers) with a gender distribution of 54.8% females. The age range of the participants was 40–73 years (mean ± SD: 56.8 ± 8.0). These individuals attended 22 assessment centers across the United Kingdom to collect their body index, encompassing a range of diverse settings to ensure socioeconomic and ethnic diversity, as well as a blend of urban and rural environments. In order to ensure comparability of lifestyle factors and biomarkers across genders, individuals of non-European ancestry were excluded. Furthermore, individuals without medical record linkage were excluded from the analysis. Baseline assessment was conducted among 502,505 out of approximately 9.2 million people invited. In our analysis, two non-APOE4 carriers for each APOE4 carrier were matched by age (± 1 year) and sex. Our study adhered to the AGReMA guidelines.

### Ascertainment of incident dementia

Dementia/AD was defined using hospital inpatient records and mortality register data with a primary/secondary diagnosis based on the international classification diseases codes (Table S1) [20]. Dementia was identified as an underlying or contributory cause of death by linking to data from the death register. Dementia diagnosed before the age of 65 was classified as young-onset dementia, while diagnoses at or after 65 years were considered late-onset dementia. The onset date of dementia refers to the earliest recorded date. Person-years were calculated from baseline assessment date to the date of onset of dementia, date of death, or the end of follow-up (31 December 2020 for England and Wales and 31 January 2021 for Scotland), whichever came first.

### Genetic data

BiLEVE Axiom array, or the UK Biobank Axiom array was used for genotyping by Affymetrix. Before the data release, genotype imputation using the Haplotype Reference Consortium reference panel was conducted by the UK Biobank team. APOE4 genotype was directly genotyped using two single-nucleotide polymorphisms (rs7412/rs429358). APOE4+ dominant model of E3/E4 or E4/E4 was used to define APOE4.

### Sociodemographic data

Age, sex, ethnicity, education, and income were self-reported. Townsend index of material deprivation was used to assess neighbourhood-level socioeconomic status.

### Lifestyle factors

A questionnaire on a touch-screen computer about lifestyle factors including diet, smoking, sleep duration, and frequency of alcohol consumption was completed. A short form of the International Physical Activity Questionnaire was used to estimate excess metabolic equivalent (MET)-hours/week of physical activity during work and leisure time. A healthy diet score was calculated based on seven commonly eaten food groups and a higher score is associated with a lower risk of dementia [20]. Participants were asked to report their average sleep duration per day over the past 4 weeks using the survey question, “About how many hours of sleep do you get in every 24 h?” Alcohol consumption and supplement intake, including vitamins, folate, glucosamine, calcium, zinc, iron, and selenium, were self-reported on a weekly basis over the past year.

### Blood tests

Lipids including total cholesterol, high-density lipoprotein cholesterol (HDL-C), low-density lipoprotein cholesterol (LDL-C), and triglycerides were tested by direct enzymatic methods (Konelab, Thermo Fisher Scientific, Waltham, Massachusetts). Glycated haemoglobin (HbA1c) was measured using high-performance liquid chromatography on a Bio-Rad Variant II Turbo. Other plasma biomarkers were also measured (https://biobank.ctsu.ox.ac.uk/crystal/ukb/docs/serum_biochemistry.pdf).

### Urinary biomarker data

Sodium, potassium, microalbumin, and creatinine in urine were measured by an ion-selective electrode analysis on a Beckman Coulter AU5400 (https://biobank.ndph.ox.ac.uk/showcase/ukb/docs/urine_assay.pdf).

### Health-related conditions and risk score

Long-standing illness, disability, or infirmity, and overall health were self-reported (poor, fair, good, excellent).

Chronic conditions including hypertension, depression, heart disease, and stroke at baseline were defined using self-reported data or interviews. Additional cases of these conditions at baseline were defined using inpatient data (initial diagnosis date before baseline interview date). Body mass index (BMI) was computed based on measured weight and height, and obesity was defined as BMI ≥ 30 kg/m2. A multimorbidity score was calculated based on these 61 major diseases (Table S2) [21]. Cardiovascular Risk Factors, Aging, and Incidence of Dementia (CAIDE) risk score [22], and Framingham Heart Study (FRS) score [23] for dementia with good prediction performance were also calculated.

### Familial medical history

The family history (father, mother, and siblings) of eight medical conditions including heart disease, stroke, hypertension, diabetes, dementia, Parkinson’s disease, and depression was collected using a touchscreen computer.

### Environment measures

Air pollution and local environment measured by the Small Area Health Statistics Unit (http://www.sahsu.org/) were linked centrally to UK Biobank data. Air pollutants including particulate matter, nitrogen dioxide, and total nitrogen oxides as annual average values in μg/m^3^ were measured. Road traffic measures to the local road network were estimated based on surrounding monitored links. Data on noise pollution, such as daytime, evening, and night-time average level of noise pollution (dB) were also available.

### Statistical analysis

Baseline characteristics by APOE4 were expressed as frequency (percentage) and means ± standard deviations (SDs). T-test for continuous variables and Chi-square test for categorical variables were used to examine the difference between APOE4 and non-APOE4 carriers.

APOE4 effects on the incidence of all-cause dementia/AD were estimated using Cox proportional hazards regression models. Whether APOE4 modified associations between a wide range of individual factors and incident all-cause dementia/AD was tested using Cox proportional hazards regression models. We tested two models: (1) Model 1 was adjusted for age and sex; (2) Model 2 was adjusted for age, sex, education, household income, BMI, smoking, physical activity, diet score, alcohol consumption, and sleep duration. Moderation analysis included socioeconomic factors (*n* = 3), lifestyle factors (*n* = 19), risk scores (*n* = 3), health-related conditions (*n* = 2), familial history of medical conditions (*n* = 24), blood biomarkers (*n* = 49), urinary biomarkers (*n* = 4), and pollution measures (*n* = 30, Table S3).

Population attributable risk (PAR) for each risk factor was computed using the formula:

$$PAR=\frac{{P}_{r}\times (HR-1)}{{1+P}_{r}\times (HR-1)}$$

where P_r_ refers to the prevalence of the risk factor and HR refers to the adjusted HR for incident dementia associated with the corresponding risk factor in Model 2. We also calculated a combined PAR using the formula: combined PAR = 1 – (1 − PAR_1_) × (1 − PAR_2_) × (1 − PAR_3_) [24]. Covariates in Model 2 and all other leading risk factors were adjusted for to estimate the HR for dementia associated with each risk factor in this analysis.

A sensitivity analysis was conducted to examine whether APOE4 modified the association between the important determinants and incident dementia by excluding those dementia cases developed in the first 5 years of follow-up. Another sensitivity analysis was conducted to test moderation associations among individuals with complete data.

Multiple imputations for missing data were conducted, and age, sex, and all covariates were included in the imputation models to create 5 imputed datasets.

Data analyses were conducted using SAS 9.4 for Windows (SAS Institute Inc.) and all *P* values were two-sided with statistical significance set at < 0.05.

## Results

### Population selection

We excluded participants of non-European ancestry (*n* = 30,380), those who could not be linked to inpatient data (*n* = 27), those with prevalent dementia (*n* = 345), or cognitive impairment (*n* = 232), or those who developed dementia in the first year of follow-up (*n* = 36). Among 459,199 participants with complete APOE4 data, 128,137 participants who were not matched in pairs were excluded. We included 331,062 (110,354 APOE4 carriers, 220,708 age- and sex-matched non-APOE4 carriers) participants (54.8% females) aged 40–73 years (mean ± SD: 56.8 ± 8.0) in the final analysis (Fig. S1).

### Baseline characteristics

APOE4 carriers were more likely to be non-current smokers, be physically inactive and have higher diet score compared with age- and sex-matched controls. APOE4 presence was associated with a higher level of triglycerides (*P* value < 0.0001), total cholesterol (*P* value < 0.0001), and LDL-C (*P* value < 0.0001), and lower HDL-C (*P* value < 0.0001) (Table 1). The father, mother, and sibling of the APOE4 carriers had a higher prevalence of dementia than those of the non-APOE4 carriers. APOE4 carriers had lower creatinine, potassium, and sodium in urine than non-APOE4 carriers (Table S4). The proportion of participants with missing values in each variable and values in imputed and non-imputed data are listed in Tables S5 and S6.

**Table 1:** Baseline characteristics by APOE4

	Non-APOE4 carrier	APOE4 carrier	*P*-value*
Age (years)	56.88 ± 8.02	56.67 ± 8.04	< 0.0001
Sex			1.00
Women	120,856 (54.8)	60,428 (54.8)	
Men	99,852 (45.2)	49,926 (45.2)	
Education			0.59
College/university degree	70,807 (32.1)	36,464 (33.0)	
Upper secondary	25,104 (11.4)	12,691 (11.5)	
Final stage of secondary education	48,144 (21.8)	23,993 (21.7)	
Lower secondary	11,908 (5.4)	5921 (5.4)	
First stage of secondary education	14,379 (6.5)	7025 (6.4)	
Vocational qualifications	11,425 (5.2)	5650 (5.1)	
None of above	38,941 (17.6)	18,610 (16.9)	
Household income (pounds)			< 0.0001
< 18,000	49,911 (22.6)	23,979 (21.7)	
18,000–30,999	60,963 (27.6)	30,298 (27.5)	
31,000–51,999	58,030 (26.3)	29,470 (26.7)	
52,000–100,000	41,411 (18.8)	21,344 (19.3)	
> 100,000	10,393 (4.7)	5263 (4.8)	
Townsend index	-1.44 ± 3.00	-1.52 ± 2.95	< 0.0001
Alcohol consumption			0.0003
Never	7314 (3.3)	3474 (3.1)	
Previous	7873 (3.6)	3745 (3.4)	
Current	205,521 (93.1)	103,135 (93.5)	
Smoking			< 0.0001
Never	119,090 (54.0)	60,292 (54.6)	
Former	78,030 (35.4)	39,831 (36.1)	
Current	23,588 (10.7)	10,231 (9.3)	
Physical activity (MET-minutes/week)	2659 ± 2441	2683 ± 2448	0.0074
Diet score^a^	3.87 ± 1.43	3.92 ± 1.43	< 0.0001
Sleep duration (hours)	7.17 ± 1.10	7.16 ± 1.08	0.0153
BMI (kg/m^2^)	27.45 ± 4.74	27.26 ± 4.67	< 0.0001
Overall health rating			< 0.0001
Excellent	36,703 (16.6)	18,687 (16.9)	
Good	128,531 (58.2)	65,068 (59.0)	
Fair	45,994 (20.8)	22,101 (20.0)	
Poor	9480 (4.3)	4498 (4.1)	
Long-standing illness, disability or infirmity			< 0.0001
No	148,184 (67.1)	75,033 (68.0)	
Yes	72,524 (32.9)	35,321 (32.0)	
Multimorbidity risk score	0.27 ± 0.29	0.26 ± 0.29	0.0835
Trunk fat percentage	31.23 ± 8.00	30.96 ± 7.99	< 0.0001
Whole body fat percentage	31.51 ± 8.52	31.25 ± 8.50	< 0.0001
HbA1c (mmol/mol)	14.19 ± 1.22	14.17 ± 1.22	0.0001
Triglycerides (mmol/L)	1.74 ± 0.98	1.79 ± 1.04	< 0.0001
Total cholesterol (mmol/L)	5.67 ± 1.13	5.86 ± 1.17	< 0.0001
HDL-C (mmol/L)	1.46 ± 0.36	1.44 ± 0.35	< 0.0001
LDL-C (mmol/L)	3.53 ± 0.84	3.69 ± 0.87	< 0.0001

Data are means ± standard deviations, or N (%)

*APOE4* Apolipoprotein E4, *BMI* Body mass index, *HbA1c* Glycated haemoglobin, *HDL-C* High-density lipoprotein cholesterol, *LDL-C* Low-density lipoprotein cholesterol, *MET* Metabolic equivalent

^*^T-test for continuous variables and Chi-square for categorical variables were used to test the difference between APOE4 and non-APOE4 carriers. APOE4+ dominant model of E3/E4 and E4/E4 was used to define the presence of APOE4

^a^Diet score was computed based on seven commonly eaten food groups following recommendations on dietary priorities for cardiometabolic health with a higher score representing a healthier diet

### Incidence of dementia

**Fig. 2:**
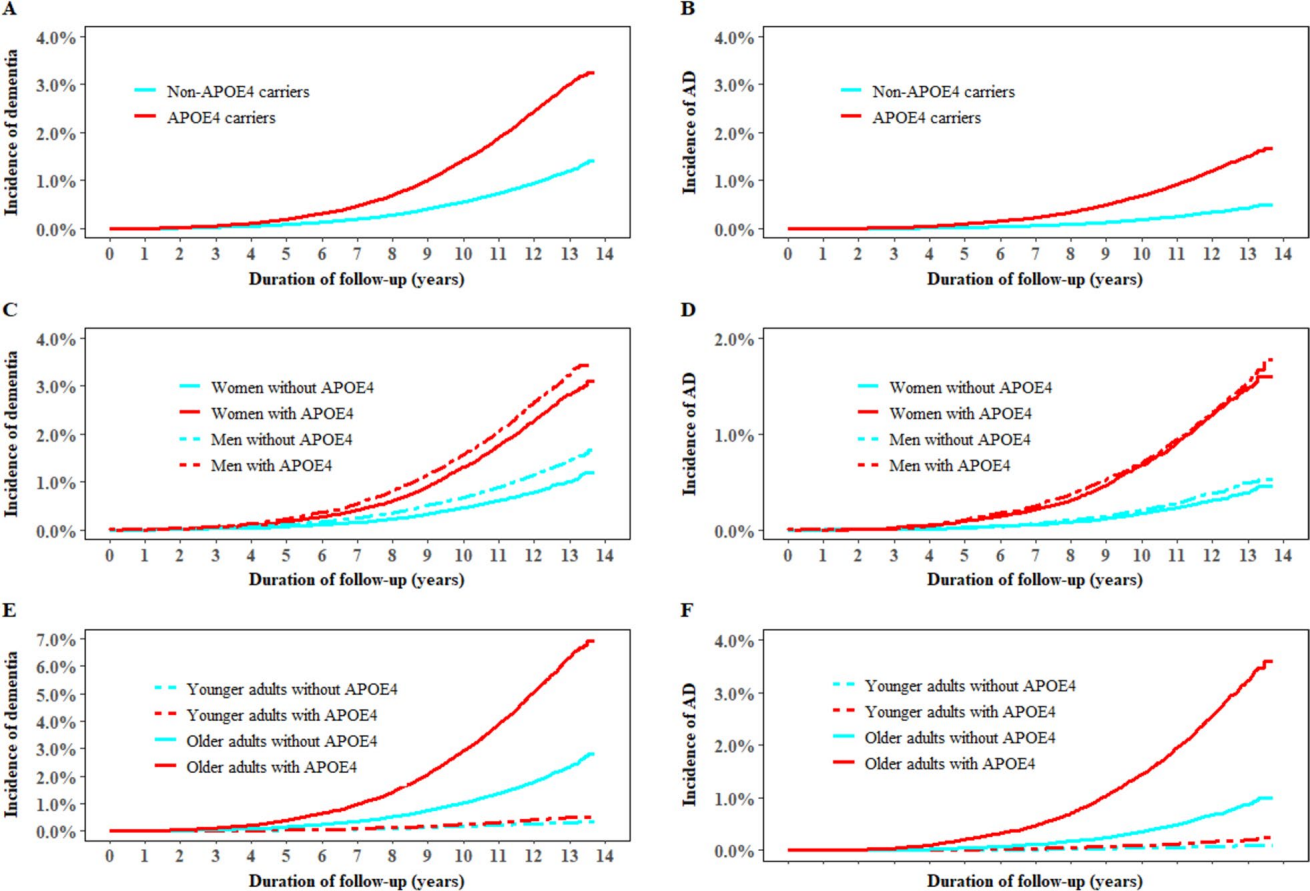
Incidence of dementia and Alzheimer’s disease in individuals with and without APOE4. AD, Alzheimer’s disease; APOE4, apolipoprotein E ε4. Panel **A** shows the incidence of all-cause dementia in individuals with and without APOE4; Panel **B** shows the incidence of Alzheimer’s disease in individuals with and without APOE4; Panel **C** shows the incidence of all-cause dementia in women and men by APOE4; Panel **D** shows the incidence of Alzheimer’s disease in women and men by APOE4; Panel **E** shows the incidence of all-cause dementia in younger and older individuals by APOE4; Panel **F** shows the incidence of Alzheimer’s disease in younger and older individuals by APOE4. Younger age was defined as < 60 years and older age as ≥ 60 years

During a median follow-up of 11.9 years (interquartile range: 11.2–12.6), 4764 incident all-cause dementia cases and 2065 incident AD cases were documented. The adjusted hazard ratios ([HRs] 95% CIs) for all-cause dementia and AD associated with APOE4 were 2.70 (2.55–2.85) and 3.72 (3.40–4.07), respectively. The association between sex and all-cause dementia was stronger in non-APOE4 carriers (HR (95% CI) for women versus men: 1.44 (1.33–1.57)) than in APOE4 carriers (1.16 (1.07–1.25), *P*-value for interaction < 0.0001). The HR (95% CI) for all-cause dementia associated with older age was larger in APOE4 carriers (HR (95% CI) for ≥ 60 years versus < 60 years: 3.92 (3.57–4.31)) than in non-APOE4 carriers (2.22 (1.67–2.97), *P*-value for interaction = 0.0003). Similar results were seen for AD (Fig. 2).

### Leading determinants for dementia

**Fig. 3:**
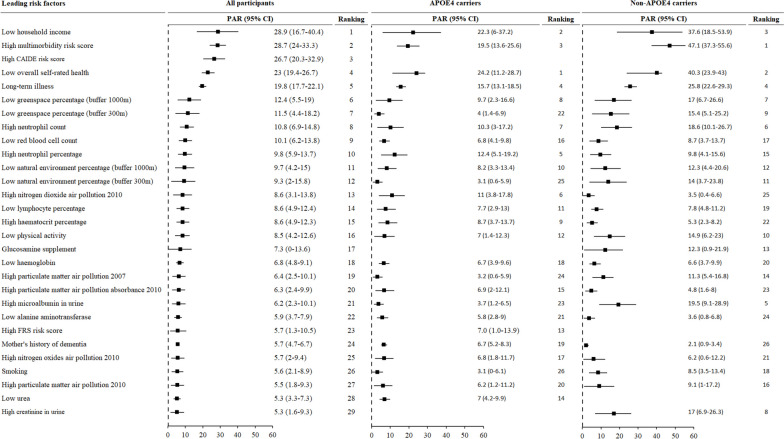
Leading risk factors for all-cause dementia in the whole population, APOE4 carriers, and non-APOE4 carriers. APOE4, apolipoprotein E ε4; CAIDE, Cardiovascular Risk Factors, Aging, and Incidence of Dementia; CI, confidence interval; PAR, population attributable risk. PAR was calculated based on the hazard ratio for dementia adjusted for age, sex, education, household income, BMI, smoking, physical activity, diet score, alcohol consumption, and sleep duration

As shown in Fig. 3, the five leading risk factors for all-cause dementia included low household income, high multimorbidity risk score, high CAIDE risk score, low overall self-rated health, and long-term illness. We calculated a multimorbidity risk score for dementia by considering the statistically significant associations between individual diseases and dementia. The score was computed using the formula: ${\sum }_{i}^{1}\mathrm{\beta i}$, where βi represents the coefficient (log (hazard ratio [HR])) for incident dementia associated with the i disease. Additionally, we also computed a multimorbidity risk score that incorporated age/APOE4. Low greenspace percentage, low natural environment percentage, or high nitrogen dioxide air pollution 2010 were also among the leading risk factors for dementia. The CAIDE risk score was not a significant risk factor for dementia in either APOE4 carriers or non-APOE4 carriers possibly because CAIDE risk score was calculated based on APOE4, age, and sex.

In APOE4 carriers, the leading risk factors for all-cause dementia included low self-rated overall health (PAR (95% CI): 24.2% (11.2–28.7%)), low household income (22.3% (6–37.2%)), high multimorbidity risk score (19.5% (13.6–25.6%), long-term illness (15.7% (13.1–18.5%)), high neutrophil percentage (12.4% (5.1–19.2%)), high nitrogen dioxide air pollution 2010 (11 (3.8–17.8)), and high neutrophil count (10.3 (3–17.2)).

In non-APOE4 carriers, the leading risk factors for all-cause dementia included high multimorbidity risk score (PAR (95% CI): 47.1% (37.3–55.6%)), low overall self-rated health (40.3% (23.9–43.0%)), low household income (37.6% (18.5–53.9%)), long-term illness (25.8% (22.6–29.3%)), high microalbumin in urine (19.5% (9.1–28.9%)), high neutrophil count (18.6% (10.1–26.7%)), low greenspace percentage (17.0% (6.7–26.6%)), and high creatinine in urine (17.0% (6.9–26.3%)).

PAR for these individual risk factors combined was 77.5%, 65.1%, and 85.8% in the whole population, APOE carriers, and non-APOE4 carriers, respectively.

### Moderation analysis for all-cause dementia

APOE4 was a significant moderator for the association between 22 individual risk factors and incident dementia. For 20 risk factors, associations with incident dementia were stronger in non-APOE4 carriers than in APOE4 carriers. For example, the HR (95% CI) for incident all-cause dementia associated with multimorbidity risk score was larger in non-APOE4 carriers (quintile 5 versus quintile 1: 3.60 (3.00–4.33)) than in APOE4-carriers ((2.02 (1.76–2.33), *P*-value for interaction < 0.0001). The corresponding number for self-rated overall health (poor versus excellent) was 4.81 (3.95–5.86) and 2.61 (2.18–3.13), respectively. Smoking and low diet quality were risk factors for all-cause dementia in non-APOE4 carriers (HR (95% CI): 1.50 (1.31–1.72) for smoking, 1.18 (1.05–1.32) for low diet quality) but not in APOE4 carriers (1.05 (0.91–1.22) for smoking, 1.04 (0.94–1.15) for low diet quality, *P*-values for interaction < 0.05). The association between particulate matter air pollutants and incident dementia was stronger in non-APOE4 carriers (quintile 5 versus quintile 1: 1.41 (1.22–1.61)) than in APOE4 carriers (1.17 (1.03–1.32), *P*-value for interaction = 0.0141).

**Fig. 4:**
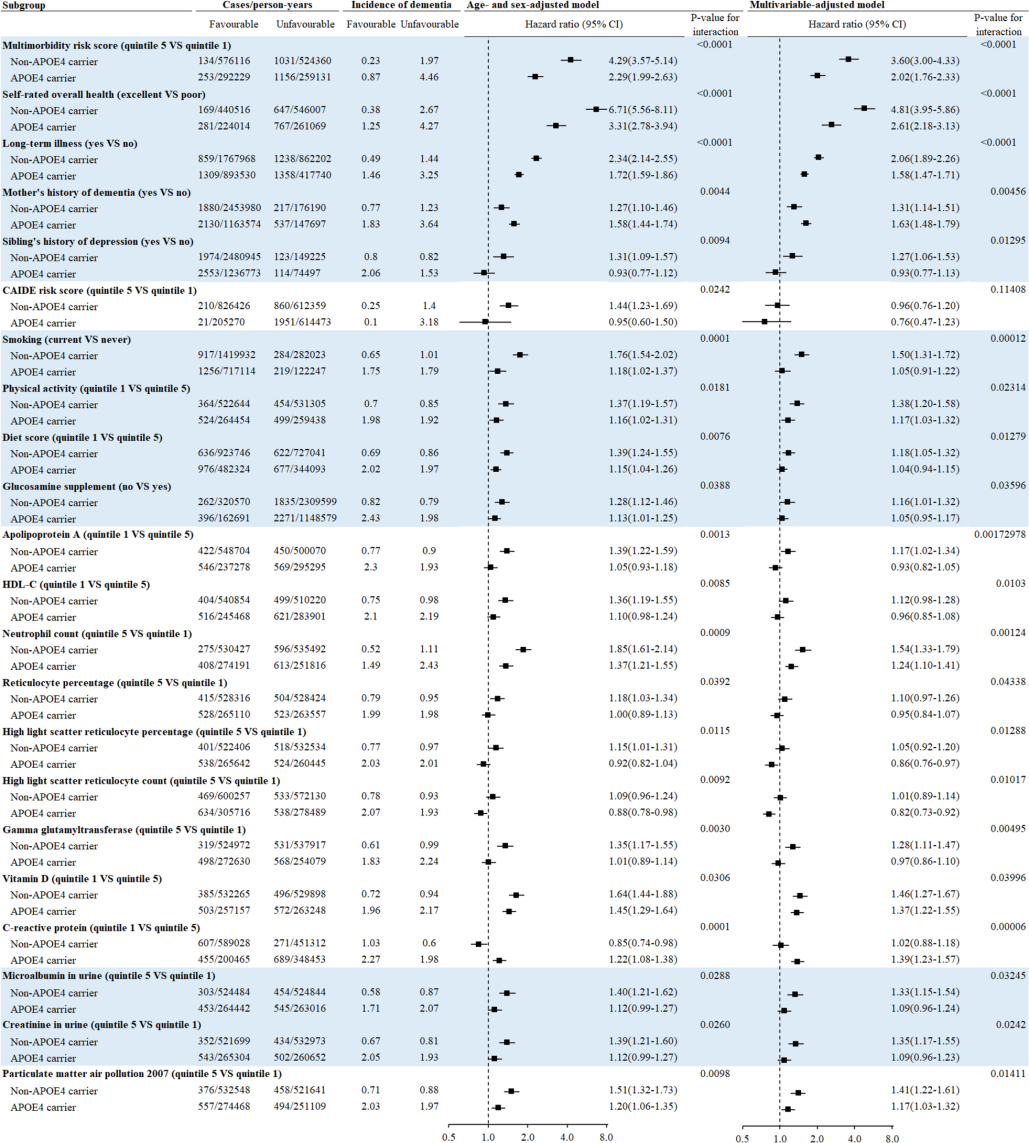
The association between leading risk factors and incident all-cause dementia stratified by APOE4. APOE4, apolipoprotein E ε4; CAIDE, Cardiovascular Risk Factors, Aging, and Incidence of Dementia; HDL-C, high-density lipoprotein cholesterol. Cox proportional hazards regression models were used to test whether APOE4 modified associations between leading risk factors and incident dementia. Multivariable model was adjusted for age, sex, education, household income, BMI, smoking, physical activity, diet score, alcohol consumption, and sleep duration

For only 2 risk factors, their associations with incident all-cause dementia were stronger in APOE4 carriers than in non-APOE4 carriers. The HR (95% CI) for dementia associated with mother’s history of dementia was 1.31 (1.14–1.51) in non-APOE4 carriers and 1.63 (1.48–1.79) in APOE4 carriers (*P*-value for interaction = 0.0046). The HR (95% CI) for dementia associated with C-reactive protein (quintile 1 versus quintile 5) was 1.02 (0.88–1.18) in non-APOE4 carriers and 1.39 (1.23–1.57) in APOE4 carriers (*P*-value for interaction = 0.0001, Fig. 4).

For other important risk factors including household income, greenspace percentage, and natural environment percentage, their associations with dementia did not differ between individuals with and without APOE4 (Figs. S2 and S3).

### Moderation analysis for Alzheimer’s disease

**Fig. 5:**
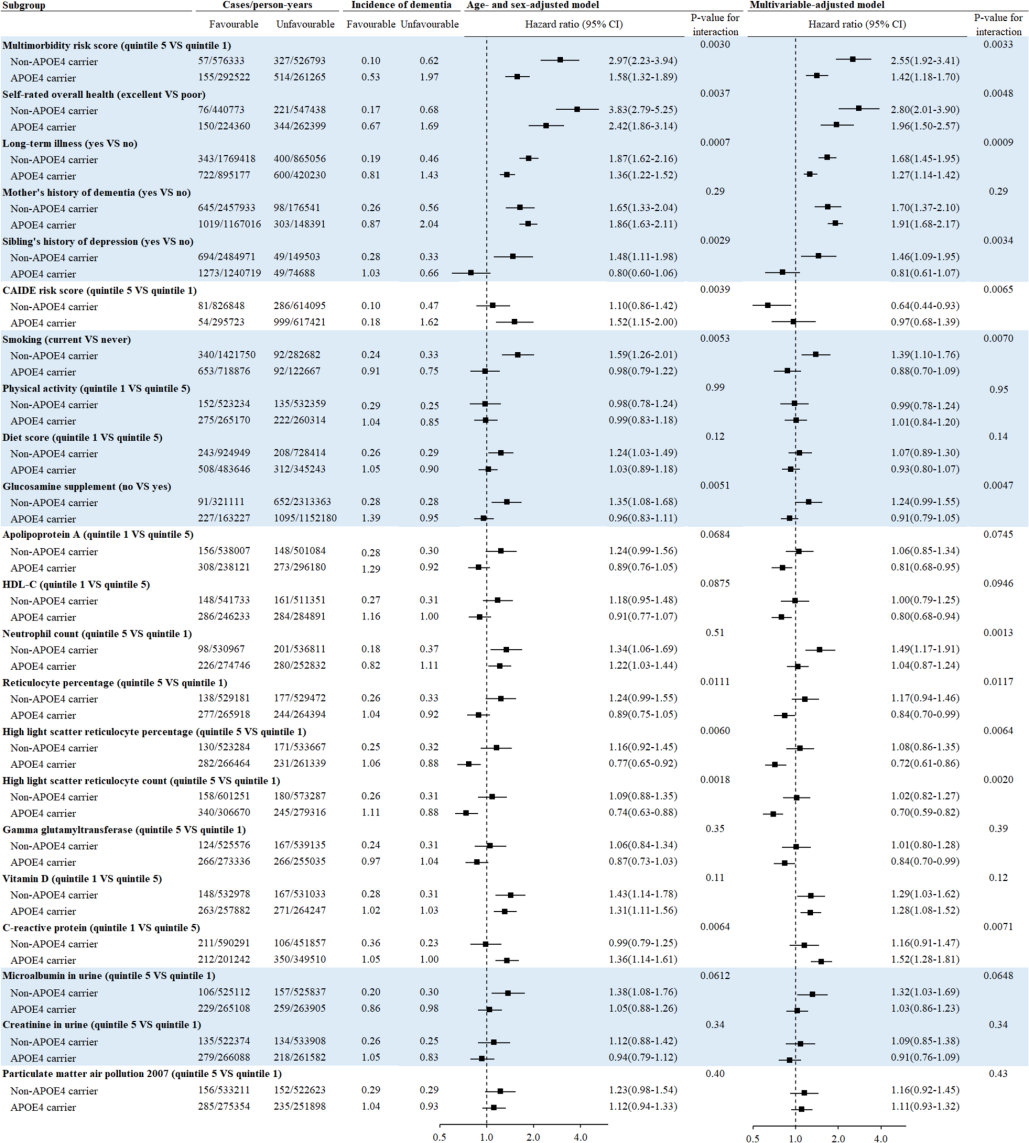
The association between leading risk factors and incident Alzheimer’s disease stratified by APOE4. APOE4, apolipoprotein E ε4; CAIDE, Cardiovascular Risk Factors, Aging, and Incidence of Dementia; HDL-C, high-density lipoprotein cholesterol. Cox proportional hazards regression models were used to test whether APOE4 modified associations between leading risk factors and incident Alzheimer’s disease. Multivariable model was adjusted for age, sex, education, household income, BMI, smoking, physical activity, diet score, alcohol consumption, and sleep duration

The association between multimorbidity risk score, self-rated overall health, and long-term illness and incident AD was stronger among non-APOE4 carriers than in APOE4 carriers. Smoking was associated with an increased risk of incident AD in non-APOE4 carriers (HR (95% CI): 1.39 (1.10–1.76)) but not in APOE4 carriers (0.88 (0.70–1.09), *P*-value for interaction = 0.0070). Lower C-reactive protein was associated with a higher risk of incident AD in APOE4 carriers (HR (95% CI) for quintile 1 versus quintile 5: 1.52(1.28–1.81)) but not in non-APOE4 carriers (1.16 (0.91–1.47), *P*-value for interaction = 0.0071, Fig. 5).

### Sensitivity analysis

When moderation analysis was conducted in individuals aged ≥ 60 years, the association with incident dementia or AD was stronger for most risk factors in non-APOE4 carriers than in APOE4 carriers but weaker for a mother’s history of dementia and C-reactive protein (Figs. S4 and S5). Similar results were seen when the moderation analysis was conducted among individuals by excluding dementia cases developed in the first 5 years of follow-up (Figs. S6 and S7) or those with complete data (Figs. S8 and S9).

## Discussion

In this large cohort study, we found APOE4 carriers had a higher risk of all-cause dementia and AD than age- and sex-matched non-APOE4 carriers. The leading determinants for all-cause dementia/AD differed between APOE4 and non-APOE4 carriers. For most factors including multimorbidity risk score, self-rated overall health, smoking, physical activity, diet quality, HDL-C, neutrophil count, vitamin D, and particulate matter air pollutants, their associations were stronger in non-APOE4 carriers than in APOE4 carriers. For only 2 risk factors (mother’s history of dementia and C-reactive protein), their associations with incident dementia were stronger in APOE4 carriers than in non-APOE4 carriers.

The importance of socioeconomic status in the development of dementia has been highlighted in previous studies [4, 25, 26]. We found household income, education, and Townsend index were all associated with incident dementia, but only low household income was among the leading risk factors. This is consistent with a recent cohort study showing that low household income was associated with an increased risk of dementia independent of education [25]. As a measure of socioeconomic inequality, self-rated overall health should not be overlooked. Self-rated overall health reflects the perception of the biological and psychological status of individuals in given cultural and social circumstances [27]. Self-rated overall health was among the five leading risk factors in individuals with and without APOE4 in our study. Chronic conditions such as hearing impairment, diabetes, hypertension, obesity, and depression have been demonstrated to be important contributors to dementia [4]. In our analysis, multimorbidity risk score created based on these chronic conditions was the leading contributor among non-APOE4 carriers and the third leading contributor among APOE4 carriers. Recent evidence has demonstrated the important role of environmental measures in mental health [28, 29]. Nine environmental measures including greenspace, natural environment, and air pollution were among the leading determinants for dementia in our study. Inflammation and immune-related markers have been linked to dementia in previous studies [30, 31]. Likely, we found high neutrophil count and low lymphocyte percentage with PAR > 5.0% were among the leading risk factors. CAIDE risk score was the third leading risk factor for dementia among the whole population but not a significant risk factor among APOE4 carriers or non-APOE4 carriers. This may be because APOE4 was involved in the calculation of the CAIDE risk score.

Evidence has shown that more than half of AD is attributable to APOE4 [10], thus it is imperative to identify leading risk factors for APOE4 carriers. Our moderation analysis demonstrated that a mother’s history of dementia and low levels of C-reactive protein were more important risk factors of dementia in APOE4 carriers than in non-APOE4 carriers. Family history represents a combination of both genetic factors and shared environmental factors including lifestyle habits and socioeconomic status among family members. Although interaction analysis was not conducted, a population study showed that family history of AD only and family history and APOE4 combined may potentially result in functional differences in episodic memory-related regions [32]. In line with our study, a cross-sectional analysis found that APOE4 was associated with lower paired-associates learning scores in individuals with family history of dementia [33]. We also found that the association between fathers’ or siblings’ history of dementia and incident dementia did not significantly differ between individuals with and without APOE4. This suggests that the mother might have a higher impact on the lifestyle habits and other environments of the family and mothers with dementia than other family members with dementia were more likely to bright out unhealthy environments in the family thus resulting in a higher risk of dementia, especially among APOE4 carriers. Inconsistent findings from previous studies regarding the association between circulating C-reactive protein and AD are observed. Data from Norway’s Nord-Trøndelag Health Study showed that higher C-reactive protein was associated with an increased risk of AD (odds ratio (95% CI): 2.37 (1.01–5.58)) in individuals aged 60–70.5 years and a decreased risk in those aged 70.6–94 years (0.39 (0.19–0.84)) [34]. A Mendelian Randomization study revealed that higher C-reactive protein was a causal risk factor for schizophrenia, coronary artery disease, and inflammatory bowel disease which are well-known risk factors for dementia [35]. The rise in levels of C-reactive protein may be a response to the disease process rather than a cause for dementia/AD. Recent research has shown that APOE4 carriers had lower levels of C-reactive protein than non-APOE4 carriers [36]. However, it is unclear why the association between C-reactive protein and incident dementia was stronger in APOE4 carriers than in non-APOE4 carriers.

For other risk factors, their associations with dementia/AD were stronger in non-APOE4 carriers than in APOE4 carriers in our analysis. We found smoking, unhealthy diet, and physical inactivity were associated with an increased risk of dementia in non-APOE4 carriers but not in APOE4 carriers suggesting modification of these lifestyle factors may be more effective for the prevention of dementia in non-APOE4 carriers. Health-related conditions including multimorbidity risk score, self-rated overall health, and long-term illness were more predictive of dementia in non-APOE4 carriers than in APOE4 carriers. A recent cohort study did not find a significant interaction between cardiometabolic multimorbidity and genetic risk score for incident dementia [37], however, APOE4 was not tested in the interaction analysis. No significant interaction between APOE4 and multimorbidity for dementia was observed in another cohort study [38], probably because of the small sample size. We also found biomarkers including neutrophil count, lymphocyte percentage, vitamin D, and microalbumin in urine as well as air pollution were stronger predictors of dementia in non-APOE4 carriers than in APOE4 carriers. Thus, the management of these risk factors may be more favorable for non-APOE4 carriers. Our findings provide new evidence on prevention and screening strategies for dementia in individuals with and without APOE4.

To our knowledge, this is the first study to investigate the leading risk factors for dementia in individuals with and without APOE4 and whether APOE4 modified associations between a wide range of risk factors and incident dementia. The study has several potential limitations. Firstly, the definition of dementia may have underestimated the incidence as the medical records or death registers may fail to identify all cases. However, it has shown that there is good agreement between case ascertainment and primary care records [39]. Secondly, given the prodromal period of dementia can last decades [40], some cases might have occurred years before the diagnosis. However, similar results between sensitivity analysis by excluding dementia cases diagnosed in the first 5 years of follow-up and the main findings were seen, which may have reduced the possibility of reverse relationships. Finally, the analysis was restricted to individuals of European ancestry in the UK Biobank cohort, which may reduce the generalizability of our findings to other ethnic groups.

In conclusion, leading risk factors for dementia/AD differed between individuals with and without APOE4. The leading determinants for all-cause dementia/AD differed between APOE4 and non-APOE4 carriers. For most factors including multimorbidity risk score, self-rated overall health, smoking, physical activity, diet quality, HDL-C, neutrophil count, vitamin D, and particulate matter air pollutants, their associations were stronger in non-APOE4 carriers than in APOE4 carriers. For only 2 risk factors (mother’s history of dementia and C-reactive protein), their associations with incident dementia were stronger in APOE4 carriers than in non-APOE4 carriers.. Our findings provide evidence for more personalized preventative approaches to dementia in APOE4 and non-APOE4 carriers.

### Supplementary Information

**Additional file 1: Figure S1.** Population selection from the UK Biobank cohort. **Figure S2.** The association between other leading risk factors with contribution ≥ 5% and incident all-cause dementia stratified by APOE4. **Figure S3.** The association between risk factors with contribution < 5% and incident all-cause dementia stratified by APOE4. **Figure S4.** The association between leading risk factors and incident all-cause dementia stratified by APOE4 in individuals aged 60 years older. **Figure S5.** The association between leading risk factors and incident Alzheimer’s disease stratified by APOE4 in individuals aged 60 years older. **Figure S6.** The association between leading risk factors and incident all-cause dementia stratified by APOE4 by excluding dementia cases developed in the first five years of follow-up. **Figure S7.** The association between leading risk factors and incident Alzheimer’s disease stratified by APOE4 by excluding dementia cases developed in the first five years of follow-up. **Figure S8.** The association between leading risk factors and incident all-cause dementia stratified by APOE4 in individuals with complete data. **Figure S9.** The association between leading risk factors and incident Alzheimer’s disease stratified by APOE4 in individuals with complete data. **Table S1.** Codes for international classification disease and self-reported fields for dementia. **Table S2.** Chronic conditions used to create the multimorbidity score for dementia. **Table S3.** Factors tested in the moderation test. **Table S4.** Other baseline characteristics in APOE4 and non-APOE4 carriers. **Table S5.** Categorical variables in imputed and non-imputed data. **Table S6.** Continuous variables in imputed and non-imputed data.

## Data Availability

Data are available in a public, open access repository (https://www.ukbiobank.ac.uk/). All methods carried out in the study were performed in accordance with relevant guidelines and regulations.
